# Clinical presentation of invasive disease caused by *Neisseria meningitidis* serogroup Y in Sweden, 1995 to 2012

**DOI:** 10.1017/S0950268817000929

**Published:** 2017-05-08

**Authors:** O. SÄLL, B. STENMARK, M. GLIMÅKER, S. JACOBSSON, P. MÖLLING, P. OLCÉN, H. FREDLUND

**Affiliations:** 1 Department of Infectious Diseases, School of Medical Sciences, Örebro University, SE-701 82 Örebro, Sweden; 2 Department of Laboratory Medicine, School of Medical Sciences, Örebro University, SE-701 82 Örebro, Sweden; 3 Department of Infectious Diseases, Karolinska University Hospital, Karolinska Institutet, Stockholm, Sweden

**Keywords:** Invasive meningococcal disease, meningococcal disease, *Neisseria meningitidis*, *Neisseria meningitidis* serogroup Y, Sweden

## Abstract

Over the period 1995–2012, the incidence of invasive meningococcal disease (IMD) caused by *Neisseria meningitidis* serogroup Y (NmY) increased significantly in Sweden. This is mainly due to the emergence of a predominant cluster named strain type YI subtype 1, belonging to the ST-23 clonal complex (cc). The aim of this study was to examine the clinical picture of patients with invasive disease caused by NmY and to analyse whether the predominant cluster exhibits certain clinical characteristics that might explain the increased incidence. In this retrospective observational study, the medical records available from patients with IMD caused by Nm serogroup Y in Sweden between 1995 and 2012 were systematically reviewed. Patient characteristics, in-hospital findings and outcome were studied and differences between the dominating cluster and other isolates were analysed. Medical records from 175 of 191 patients were retrieved. The median age was 62 years. The all-cause mortality within 30 days of admission was 9% (15/175) in the whole material; 4% (2/54) in the cohort with strain type YI subtype 1 and 11% (12/121) among patients with other isolates. Thirty-three per cent of the patients were diagnosed with meningitis, 19% with pneumonia, 10% with arthritis and 35% were found to have bacteraemia but no apparent organ manifestation. This survey included cases with an aggressive clinical course as well as cases with a relatively mild clinical presentation. There was a trend towards lower mortality and less-severe disease in the cohort with strain type YI subtype 1 compared with the group with other isolates.

## INTRODUCTION

Invasive meningococcal disease (IMD) causes significant mortality and morbidity worldwide and may present in different ways ranging from a mild febrile illness to fulminant septic shock, with or without meningitis. Nasopharyngeal colonisation and haematogenous spread precede presentation of the disease [1]. After isolation from normally sterile body fluids (i.e. blood, cerebrospinal fluid (CSF) or joint fluid) the *Neisseria meningitidis* (Nm) are further characterised serologically or genetically into capsular serogroups where A, B, C, Y and W account for the majority of cases with invasive disease.

The clinical picture of IMD has been described in previous studies where the different serogroups tend to present in different ways, with serogroups A, B and C considered causing more severe disease than serogroups W and Y [2]. Disease severity and hypervirulence are associated with specific clonal complexes (cc) disclosed by multilocus sequence typing (MLST) [3].

In previous studies, meningitis has been reported in about 50% [4] and pneumonia in 5–17% of patients with IMD [5, 6]. Serogroups W and Y are more likely to cause pneumonia compared with the other capsular serogroups [6]. However, there are difficulties when diagnosing meningococcal pneumonia, as airway cultures fail to distinguish between colonisation and organisms causing acute disease [2, 7].

The epidemiology of IMD varies dynamically from sporadic cases to local outbreaks and epidemics, as well as replacement of strains among asymptomatic carriers [8]. The incidences of IMD in Europe, North America and other economically developed areas vary [9] and historically have been relatively low with overall incidences ranging between 0.2 and 3 cases per 100 000 inhabitants per year; mainly caused by serogroups B or C [10]. Over the last 20 years the proportion of cases with *N. meningitidis* serogroup Y (NmY) has increased in several countries including the USA, Sweden, Finland, Norway, Colombia and Venezuela [4, 5, 11–13]. In 2012, the highest relative proportion (49%) of serogroup Y IMD in Europe was found in Sweden, as well as the highest incidence of serogroup Y IMD (0.46/100 000 inhabitants from total IMD incidence of 1/100 000) [14, 15]. Meningococcal vaccination has never been a part of the routine vaccination programme in Sweden.

**Fig. 1.**
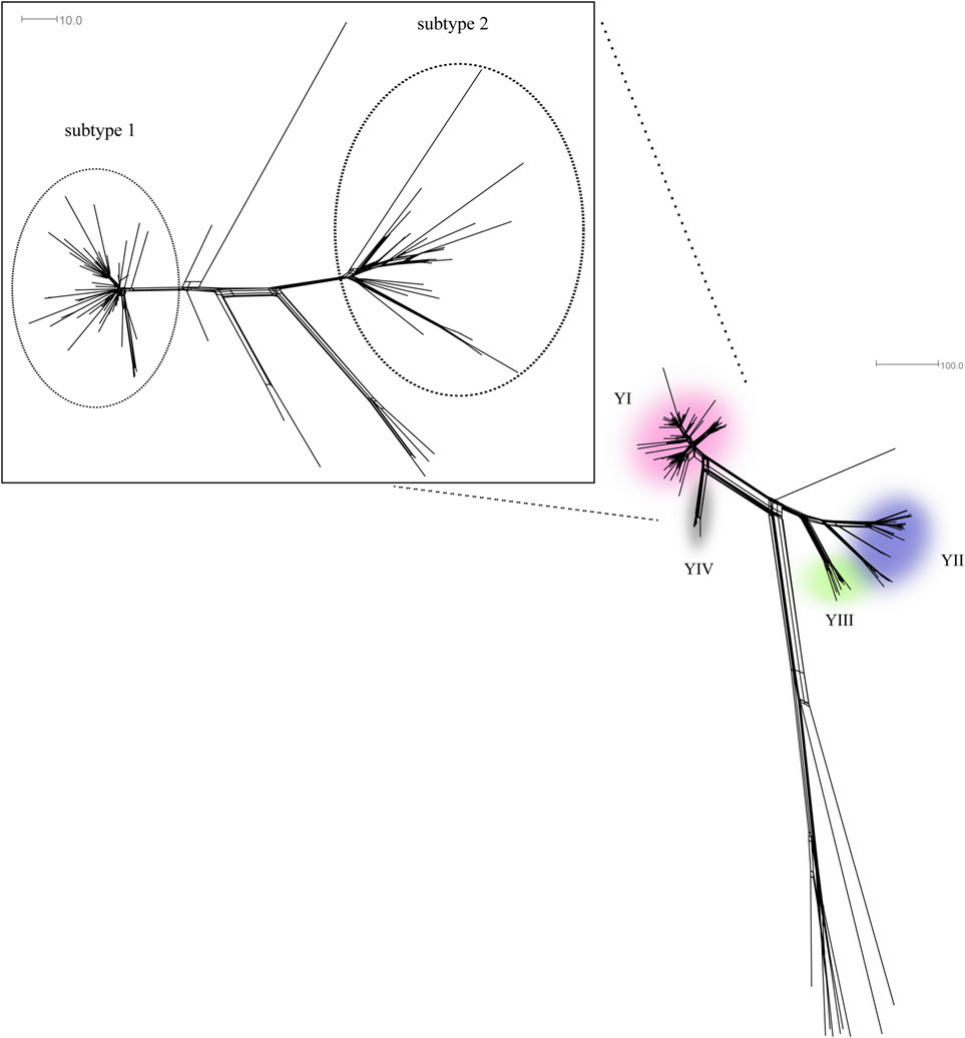
Neighbour-Net graph constructed with 1241 of 1600 core genes, as defined in the PubMLST database, analysed on isolates from patients with NmY IMD in Sweden 1995–2012. Isolates from the most prevalent NmY strain types (YI, YII, YII and YIV) are found in the respective coloured areas. The YI strain type is amplified to visualise the close genetic relationships between clusters, where the dominating clusters subtype 1 and subtype 2 have been encircled, based on analysis of 1387 core genes. The genetic distance was defined as number of loci with allelic differences.

In previous studies all invasive isolates of NmY in Sweden 1995–2012 were characterised using WGS (whole-genome sequencing) that revealed the emergence of a strain type referred to as strain type YI belonging to the ST-23 cc. Furthermore, analysis of 1600 core genome MLST (cgMLST) genes revealed that the strain type YI could subsequently be separated into clusters where the majority of isolates (*n* = 54) was defined as a certain strain subtype given the name ‘strain type YI subtype 1’ (Fig. 1). This cluster was first seen in 2006 and was responsible for the main increase in NmY IMD in Sweden during the study period [14, 16, 17].

We here describe the epidemiology and laboratory features as well as the clinical presentation of all patients with serogroup Y IMD recognised in Sweden between 1995 and 2012, covering both low and high incidence periods.

## METHODS

A total of 191 patients with serogroup Y IMD were identified in Sweden during the 1995–2012 study period. Invasive infections caused by *N. meningitidis* according to the European Union case definition (http://eur-lex.europa.eu/legal-content/EN/TXT/PDF/?uri=CELEX:32012D0506&qid=1428573336660&from=EN) are mandatorily reported in Sweden and all clinical isolates are sent to the National Reference Laboratory for Pathogenic *Neisseria* at Örebro University Hospital for further characterisation. Capture–recapture analyses performed at our laboratory have shown that the proportion of non-captured cases is below 5% [18].

Of the 191 known episodes of serogroup Y IMD during the study period, medical records for 175 (92%) patients were retrospectively and systematically reviewed. For technical reasons, 16 medical records could not be found, the majority from 1995 to 1999.

The clinical data were gathered and recorded using a standardised questionnaire. The County Medical Officer for Communicable Disease Control and Prevention in each county reviewed the medical records from his/her respective area, and transferred clinical data from each patient to the questionnaire forms that were then sent to and later compiled by the authors.

The data collected comprised patient characteristics (e.g. age, gender and smoking) in-hospital findings (e.g. blood pressure, temperature, general appearance, kidney function, time to antibiotic treatment and lumbar puncture) as well as outcome (e.g. 30-day mortality, sequelae after 6 and 12 months, respectively, and final diagnosis). In order to further investigate the increase in incidence starting 2006, the patient cohort with strain type YI subtype 1 was compared with the group of patients with other isolates pooled together to determine whether this cluster presented with a different clinical picture. Differences in clinical and laboratory findings as well as outcome between patients with strain type YI subtype 1 and other NmY isolates were analysed.

The diagnosis pneumonia was based on a clinical picture and X-ray findings suggesting pneumonia in combination with NmY bacteraemia. Bronchoalveolar or other airway cultures were not regularly performed to confirm the diagnosis. Arthritis was recorded if either culture or polymerase chain reaction (PCR) on joint fluid was positive for NmY, with or without bacteraemia. Meningitis was recorded if CSF culture or PCR revealed NmY, with or without bacteraemia. A clinical picture of meningitis in combination with NmY bacteraemia was recorded as meningitis in those cases where lumbar puncture had been considered inappropriate and not performed. Other manifestations included any other localised infection site, based on clinical presentation combined with positive blood culture. Bacteraemia with no known focus was recorded when blood culture was positive for NmY but no end-organ manifestation was found.

Statistical analyses were performed using statistical software (IBM SPSS statistics 22.0, IBM Corporation, Armonk, NY, USA). The Pearson *χ*^2^ method was used to determine statistical significance between groups, and Fischer's exact test if sample size was small. The Clopper–Pearson method (exact binominal) was used for the calculation of confidence intervals for a sample proportion (http://epitools.ausvet.com.au/content.php?page=CIProportion).

The study was approved by the Regional Ethical Review Board in Uppsala (reference number 2014/150).

## RESULTS

**Fig. 2.**
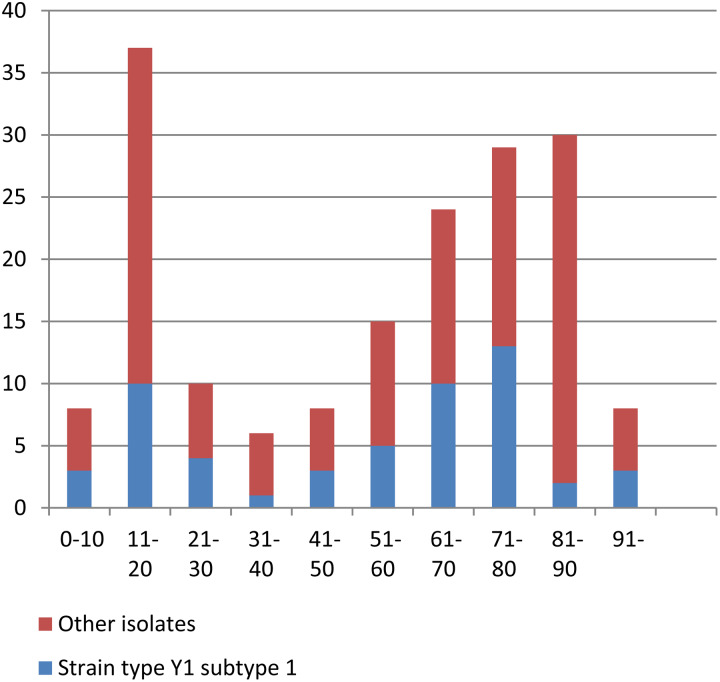
Age distribution of the 175 patients with serogroup Y IMD in Sweden 1995–2012 presented as total numbers, and also presented according to strain type YI subtype 1 and other isolates.

The median age of the 175 patients in the study was 62 years, and two distinct age groups, 11–20 years and patients older than 60 years, together represented the majority of cases (73% of all patients). Four patients were <5 years of age and only one was <1 year (Table 1, Fig. 2).

**Table 1.** Characteristics of the 175 patients with serogroup Y IMD in Sweden 1995–2012

	Total	Strain type YI subtype 1	Other isolates	*P*-value	Medical records not found
N	175	54	121		16/191
Age, years					
Median	62.0	62.0	62.0		23.0
Range	0–96	0–96	1–96		0–95
Mean	54	52	55		41
Female gender	79 (55%)	28 (52%)	68 (56%)	0.594	9/16 (56%)
Immunocompromised state*	43/171 (25%)	13/51 (25%)	30/120 (25%)	0.946	
Smoker†	18/103 (17%)	7/33 (21%)	11/70 (16%)	0.493	

* Due to haematological malignancy, alcohol abuse, diabetes, splenectomised, complement deficiency or immunosuppressive treatment with immunomodulating drugs, cytostatic drugs or corticosteroids equivalent to >7.5 mg prednisolone daily. Data not found in 4/175 patients (2%).

† Active smoker. Data not found in 72/175 patients (41%).

Two patients were not admitted but still received adequate oral antibiotic treatment. The median length of stay amongst the hospitalised patients was 9 days. There were no signs of seasonal variation in this study. All cases were sporadic apart from two brothers who became ill within 4 days of each other, both with meningitis. Two of the patients in this study, both with complement deficiency, had previously been vaccinated with ACWY vaccine.

The median time for start of antibiotic treatment was 2 h after hospital arrival and 52/141 (37%) of the patients were given antibiotics within 1 h. Data on time to antibiotics were not available in 34 patients (19%). All patients initially received antibiotics with effect on invasive meningococci except for two patients with septic arthritis who were first given empiric treatment aiming at *Staphylococcus aureus*. A brain CT scan was performed prior to lumbar puncture in 24 patients. Among these patients the median time to antibiotic start was 1 h, but the median time from admission to lumbar puncture was 13 h. When CT scan was not performed the median time to lumbar puncture was 1 h.

Meningitis was diagnosed in 33% and pneumonia in 19% of all patients (Table 2). Among other infection sites combined with bacteraemia were: epiglottitis (*n* = 5); otitis media (*n* = 2); spondylodiscitis (*n* = 2); tonsillitis (*n* = 1) and fasciitis (*n* = 1). No cases of sinusitis, conjunctivitis, urethritis, purulent pericarditis or chronic meningococcaemia were seen. Five patients were diagnosed with more than one infection focus including: meningitis and otitis media (*n* = 2); meningitis and pneumonia (*n* = 1); pneumonia and arthritis (*n* = 1); and a combination of meningitis, pneumonia and arthritis (*n* = 1). Meningitis was mainly seen among younger patients (median age 22.5 years), and the diagnosis group with the highest median age was pneumonia (77.5 years), see Table 3.

**Table 2.** Comparison of outcome between strain type YI subtype 1 cohort and the group with other isolates among the 175 patients with serogroup Y IMD in Sweden 1995–2012

	Total (*n* = 175)	Strain type YI subtype 1 (*n* = 54)	Other isolates (*n* = 121)	*P*-value
Mortality within 30 days	15 (9%) (CI 5–14%)	2 (4%) (CI 1–13%)	13 (11%) (CI 6–18%)	0.153
Intensive care unit treatment	62/173 (36%)	22/54 (41%)	40/119 (34%)	0.365
Assisted ventilation	22/174 (13%)	8/54 (15%)	14/120 (12%)	0.563
Sequelae 6 months*	23/130 (18%)	8/44 (18%)	15/86 (17%)	0.917
Sequelae 12 months†	15/125 (12%)	7/45 (16%)	8/80 (10%)	0.359
Bacteraemia with no known focus	61 (35%)	21 (39%)	40 (33%)	0.455
Meningitis	58 (33%)	19 (35%)	39 (32%)	0.701
Pneumonia	34 (19%)	8 (15%)	26 (21%)	0.303
Arthritis	17 (10%)	6 (11%)	11 (9%)	0.677
Other manifestation‡	11 (6%)	2 (4%)	9 (7%)	0.347

* Based on survivors after 6 months (*n* = 156). Data not found in 26/156 patients (17%).

† Based on survivors after 12 months (*n* = 154). Data not found in 30/154 patients (19%).

‡ Other manifestation include: epiglottitis (*n* = 5); otitis media (*n* = 2); spondylodiscitis (*n* = 2); tonsillitis (*n* = 1) and fasciitis (*n* = 1). All patients with ‘other manifestation’ had a positive blood culture except one case of spondylodiscitis with positive disc biopsy culture only.

**Table 3.** Outcome in relation to final diagnosis among the 175 patients with serogroup Y IMD in Sweden 1995–2012

	Total	Bacteraemia with no known focus	Meningitis	Pneumonia	Arthritis	Other manifestation
Age, years						
Median	62.0	65.0	22.5	77.5	72.0	62.0
Range	0–96	1–96	0–87	14–91	53–91	19–87
Mean	54	56	37	69	71	59
Mortality within 30 days	15/175 (9%)	8/61 (13%)	4/58 (7%)	3/34 (9%)	0/17 (0%)	0/11 (0%)
Intensive care unit treatment	62/173 (36%)	15/61 (25%)	36/56 (64%)	7/34 (21%)	1/16 (6%)	6/10 (60%)
Assisted ventilation	22/174 (13%)	3/61 (5%)	15/57 (26%)	1/34 (3%)	0/16 (0%)	4/11 (36%)
Sequelae 6 months	23/130 (18%)	5/42 (12%)	15/45 (33%)	1/25 (4%)	2/12 (17%)	0/7 (0%)
Sequelae 12 months	15/125 (12%)	2/41 (5%)	10/41 (24%)	0/24 (0%)	2/13 (15%)	0/7 (0%)

Based on survivors after 6 months (*n* = 156). Data not found in 26/156 patients (17%).

Based on survivors after 12 months (*n* = 154). Data not found in 30/154 patients (19%).

The clinical symptoms and findings shortly before or on admission were similar for all strain types. The patients with strain type YI subtype 1 were less prone to develop petechiae or ecchymosis compared with the other isolates. Diarrhoea, vomiting or both were seen in 40% of the patients, but there was no difference between the strain type YI subtype 1 and other isolates regarding gastrointestinal symptoms. In 31% of the patients a respiratory tract infection preceded the onset of invasive disease (Table S1 in the Supplementary Material).

Mortality within 30 days of admission was 9% (15/175) (95% CI 5–14%), with a mortality of 4% (2/54) (95% CI 1–13%) in the strain type YI subtype 1 cohort and 11% (13/121) (95% CI 6–18%) in the group with the other isolates (ns), see Table 2. Ten patients died within 5 days. The highest mortality (13%) was seen among patients with bacteraemia with no known focus (Table 3). The two patients in the strain type YI subtype 1 group who died within 30 days of admission had bacteraemia with no apparent infection focus. The other patients who died within 30 days were diagnosed with meningitis, pneumonia and bacteraemia with no known focus.

Sequelae were seen in 18% (23/130) of the patients at the 6-month follow-up and in 12% (15/125) of the patients at the 12-month follow-up. The final diagnosis most strongly associated with sequelae was meningitis with 33% (15/45) and 24% (10/41) after 6 and 12 months, respectively, (Table 2). The most frequently encountered sequelae were tiredness, hearing impairment, vertigo and cognitive impairment. Two patients with bacteraemia with no known focus developed finger necrosis which led to amputation.

## DISCUSSION

To the best of our knowledge this is the largest study describing the clinical picture of IMD caused by NmY. A wide spectrum of clinical presentations was seen ranging from mild to fulminant disease but few patients presented with low platelet count as a sign of severe disease.

*N. meningitidis* is a well-known pathogen in meningitis and sepsis, but other end-organ manifestations of IMD are also described, in particular pneumonia and septic arthritis [8, 19]. In this Swedish survey, pneumonia was diagnosed in 19% of all patients with invasive NmY disease. This proportion is slightly higher than previous reports from studies including all Nm serogroups, where pneumonia was found in 5–17% of patients [6]. The median age of patients with pneumonia (77.5 years) was clearly higher than among patients without pneumonia (58 years); this possibly being the result of patient fragility rather than specific bacterial factors. *N. meningitidis* is generally a rare cause of pneumonia [20] but when it is diagnosed it must be taken seriously due to the potential risk for secondary cases among close contacts, a situation that can be prevented by antibiotic prophylaxis.

Interestingly, five patients were diagnosed with epiglottitis. Previously most cases of epiglottitis in Sweden were caused by *Haemophilus influenzae* type B (Hib), but after the introduction of Hib vaccination, other pathogens including *N. meningitidis* have emerged as causative agents.

After arrival at the hospital, the median times to start of antibiotic treatment and performance of lumbar puncture were 2 and 3 h, respectively. These are relatively long delays and may be explained by mild symptoms at presentation that were not indicative of severe invasive infection. This is consistent with our data from the initial assessments at the emergency room, where 41% of the patients were triaged as being not severely ill, leading to delay in examination and treatment. We found that a brain CT scan prolonged the time between admission and lumbar puncture but did not delay start of antibiotic treatment. This could in part be explained by atypical presentation of meningitis.

Two distinct age groups dominated; 11–20-years old and older than 60 years, with only one infant among the patients in this study. This age distribution represents a change in meningococcal epidemiology in Sweden where IMD is now largely a disease of the elderly and with a dominance of serogroup Y. Carriage rates and serogroup distribution of *N. meningitidis* in Sweden is however unknown and further investigations in this field is needed.

Despite improvements in medical treatment and supportive care, and low meningococcal antimicrobial resistance rates, overall IMD mortality in developed countries is still around 10%, and some studies report mortality rates as high as 20% [19, 21, 22]. In this study, mortality within 30 days of admission was 9% which is consistent with previous reports. There were no significant differences in mortality or other variables studied between the strain type YI subtype 1 cohort and the group with other NmY isolates. However for many variables there was a tendency towards a milder clinical picture in the strain type YI subtype 1 cohort, suggesting that this cluster tends to be less aggressive than other isolates. The emergence of this predominant cluster in Sweden could well be the result of superior colonisation or invasive capacity. The lack of significant differences might be due to the limited size of the study material.

One limitation of the study is its retrospective design. However, it is difficult to conduct a prospective study when the overall incidence of serogroup Y IMD is low. Another limitation is the distribution of cases over time, as treatment guidelines have been modified during the study period. Finally, patients with sepsis were generally not classified to the SIRS (Systemic Inflammatory Response Syndrome) criteria and therefore distinction between severe sepsis and septic shock was not possible.

## Supplementary material

For supplementary material accompanying this paper visit https://doi.org/10.1017/S0950268817000929. click here to view supplementary material

## References

[1] van Deuren M, Brandtzaeg P, van der Meer JW. Update on meningococcal disease with emphasis on pathogenesis and clinical management. Clinical Microbiology Reviews 2000; 13: 144–166. 10627495 10.1128/cmr.13.1.144-166.2000 PMC88937

[2] Vienne P, The role of particular strains of *Neisseria meningitidis* in meningococcal arthritis, pericarditis, and pneumonia. Clinical Infectious Diseases 2003; 37: 1639–1642. 14689345 10.1086/379719

[3] Read RC. *Neisseria meningitidis*; clones, carriage, and disease. Clinical Microbiology and Infection 2014; 20: 391–395. 24766477 10.1111/1469-0691.12647

[4] Rosenstein NE, The changing epidemiology of meningococcal disease in the United States, 1992–1996. Journal of Infectious Diseases 1999; 180: 1894–1901. 10558946 10.1086/315158

[5] Racoosin JA, Serogroup Y meningococcal disease in Chicago, 1991–1997. JAMA 1998; 280: 2094–2098. 9875877 10.1001/jama.280.24.2094

[6] Vossen M, Mitteregger D, Steininger C. Meningococcal pneumonia. Vaccine 2016; 34: 4364–4370. 27443594 10.1016/j.vaccine.2016.07.013

[7] Winstead JM, Meningococcal pneumonia: characterization and review of cases seen over the past 25 years. Clinical Infectious Diseases 2000; 30: 87–94. 10619738 10.1086/313617

[8] Rosenstein NE, Meningococcal disease. New England Journal of Medicine 2001; 344: 1378–1388. 11333996 10.1056/NEJM200105033441807

[9] Gianchecchi E, *Neisseria meningitidis* infection: who, when and where? Expert Review of Anti-infective Therapy 2015; 13: 1249–1263. 26190347 10.1586/14787210.2015.1070096

[10] Halperin SA, The changing and dynamic epidemiology of meningococcal disease. Vaccine 2012; 30: B26–36. 22178525 10.1016/j.vaccine.2011.12.032

[11] Centers for Disease Control and Prevention. Prevention and control of meningococcal disease recommendations of the Advisory Committee on Immunization Practices (ACIP). Morbidity and Mortality Weekly Report 2005; 54: 1–21. 15917737

[12] Bröker M, Meningococcal serogroup Y disease in Europe: continuation of high importance in some European regions in 2013. Human Vaccine and Immunotherapeutics 2015; 11: 2281–2286. 10.1080/21645515.2015.1051276 PMC4635851 26036710

[13] Harrison LH, Antigenic shift and increased incidence of meningococcal disease. Journal of Infectious Diseases 2006; 193: 1266–1274. 16586364 10.1086/501371

[14] Törös B, Surveillance of invasive *Neisseria meningitidis* with a serogroup Y update, Sweden 2010 to 2012. Eurosurveillance 2014; 19: pii=20940. 10.2807/1560-7917.es2014.19.42.20940 25358044

[15] Bröker M, Meningococcal serogroup Y emergence in Europe: high importance in some European regions in 2012. Human Vaccine and Immunotherapeutics 2014; 10: 1725–1728. 10.4161/hv.28206 PMC5396223 24608912

[16] Thulin Hedberg S, Genetic characterisation of the emerging invasive *Neisseria meningitidis* serogroup Y in Sweden, 2000 to 2010. Eurosurveillance 2011; 16: pii=19885. 21679677

[17] Törös B, Genome-based characterization of emergent invasive *Neisseria meningitidis* serogroup Y isolates in Sweden from 1995 to 2012. Journal of Clinical Microbiology 2015; 53: 2154–2162. 25926489 10.1128/JCM.03524-14 PMC4473204

[18] Olcén P, Fredlund H. Missar vi fall av meningokockmeningit/sepsis. EPI-aktuellt, Folkhälsomyndigheten 2003; 24: 1 (Article in Swedish).

[19] Pace D, Pollard AJ. Meningococcal disease: clinical presentation and sequelae. Vaccine 2012; 30: B3–B9. 22607896 10.1016/j.vaccine.2011.12.062

[20] Bartlett JG, Mundy LM. Community-acquired pneumonia. New England Journal of Medicine 1995; 333: 1618–1624. 7477199 10.1056/NEJM199512143332408

[21] Swartz MN. Bacterial meningitis – a view of the past 90 years. New England Journal of Medicine 2004; 351: 1826–1828. 15509815 10.1056/NEJMp048246

[22] Centers for Disease Control and Prevention. Active bacterial core surveillance report, Emerging Infections Program Network, *Neisseria meningitidis*, 2014. http://www.cdc.gov/abcs/reports-findings/survreports/mening14.pdf

