# Supplementary material for: Clinical presentation of invasive disease caused by Neisseria meningitidis serogroup Y in Sweden, 1995 to 2012
Source: Epidemiol Infect. 2017 May 8;145(10):2137–43. doi: 10.1017/S0950268817000929 (PMC5968308; doi:10.1017/S0950268817000929)
Supplement: Supplementary file 1 [file S0950268817000929sup001.docx]

*Epidemiology and Infection.* Clinical presentation of invasive disease caused by *Neisseria meningitidis* serogroup Y in Sweden, 1995 to 2012. Säll O, Stenmark B, Glimåker M, Jacobsson S, Mölling P, Olcén P, Fredlund H. Supplementary Material.

Supplementary Table S1. Symptoms and findings shortly before or on admission among the 175 patients with group Y IMD in Sweden 1995-2012.

|  | **Total** | **Strain type YI subtype 1** | **Other isolates** | **p-value** | **Missing data^[[1]](#footnote-1)^** |
| --- | --- | --- | --- | --- | --- |
| **Headache** | 62/159 (39%) | 21/48 (44%) | 41/111 (37%) | 0.419 | 16 (9%) |
| **Muscle pain** | 36/160 (23%) | 9/49 (18%) | 27/111 (24%) | 0.406 | 15 (9%) |
| **Diarrhoea** | 20/163 (12%) | 7/49 (14%) | 13/114 (11%) | 0.607 | 12 (7%) |
| **Vomiting** | 61/162 (38%) | 17/49 (35%) | 44/113 (39%) | 0.609 | 13 (7%) |
| **Respiratory tract infection** | 84/165 (51%) | 28/50 (56%) | 56/115 (49%) | 0.388 | 10 (6%) |
| **Systolic blood pressure <90 mmHg** | 18/152 (12%) | 4/45 (9%) | 14/107 (13%) | 0.465 | 23 (13%) |
| **Diastolic blood pressure <60 mmHg** | 21/144 (15%) | 6/41 (15%) | 15/103 (15%) | 0.991 | 31 (18%) |
| **Pulse > 90/min** | 107/153 (70%) | 30/47 (64%) | 77/106 (73%) | 0.273 | 22 (13%) |
| **Petechiae** | 20/140 (14%) | 0/43 (0%) | 20/97 (21%) | 0.001 | 35 (20%) |
| **Ecchymosis** | 9/141 (6%) | 0/46 (0%) | 9/95 (9%) | 0.031 | 34 (19%) |
| **Elevated creatinine^[[2]](#footnote-2)^** | 54/150 (36%) | 21/49 (43%) | 33/101 (33%) | 0.223 | 25 (14%) |
| **Elevated liver enzymes^[[3]](#footnote-3)^** | 26/109 (24%) | 12/34 (35%) | 14/75 (19%) | 0.059 | 66 (38%) |
| **Respiratory rate >20/min** | 53/87 (61%) | 19/32 (59%) | 34/55 (62%) | 0.822 | 88 (50%) |
| **Neck stiffness** | 39/128 (30%) | 13/43 (30%) | 26/85 (31%) | 0.967 | 47 (27%) |
| **Affected general appearance** | 98/141 (70%) | 29/44 (66%) | 69/97 (71%) | 0.532 | 34 (19%) |
| **Decreased level of consciousness** | 42/156 (27%) | 10/49 (20%) | 32/107 (30%) | 0.214 | 19 (11%) |

1. Data not found in the medical records. [↑](#footnote-ref-1)
2. Serum creatinine above reference range adjusted for gender and age. First test, taken on admission. [↑](#footnote-ref-2)
3. Serum liver enzymes (alanine aminotransferase or aspartate aminotransferase) above reference range. First test, taken on admission. [↑](#footnote-ref-3)
